# Sacubitril/Valsartan in Patients with HFrEF Undergoing Maintenance Hemodialysis, Evidence of Cardiac Reverse Remodeling During 12-Month Follow-Up: Case Series

**DOI:** 10.3390/reports9030291

**Published:** 2026-09-01

**Authors:** Sonila Mocka, Danilo Baccino, Ledio Shurdi, Stefano Cornara, Stefano Ferraro

**Affiliations:** 1 Nephrology and Dialysis Unit, Department of Internal Medicine, San Paolo Hospital, Via Genova 30, 17100 Savona, Italy; 2 Cardiolody and Cardiovascular Intensive Care Unit, Department of Emergency Medicine, San Paolo Hospital, 17100 Savona, Italy; 3 Department of Cardiology, Vivantes Humboldt-Klinikum, 13509 Berlin, Germany

**Keywords:** sacubitril/valsartan, heart failure, left ventricular ejection fraction, dialysis, cardiac remodeling, end-stage kidney disease

## Abstract

**Background and Clinical Significance:** Heart failure with reduced ejection fraction (HFrEF) is highly prevalent among patients with end-stage kidney disease (ESKD) undergoing maintenance hemodialysis (MHD) and is associated with poor clinical outcomes. Sacubitril/valsartan (SV) is a cornerstone therapy for HFrEF; however, its use in dialysis patients remains limited due to safety concerns and lack of robust evidence. This case series describes the clinical response and safety of SV in patients with HFrEF receiving MHD; **Case Presentation:** Four male patients with HFrEF undergoing MHD were treated with SV and followed for 12 months. Baseline evaluation showed a left ventricular ejection fraction (LVEF) of 35.5%, left ventricular end-systolic volume (LVESV) of 118.5 mL, left ventricular end-diastolic volume (LVEDV) of 185.7 mL, pulmonary artery systolic pressure (PASP) of 52.7 mmHg, and NT-proBNP levels above 35,000 pg/mL in all patients. After 12 months of SV therapy, LVEF improved to 53.7%, corresponding to a 51.4% relative increase from baseline. Reverse cardiac remodeling was observed, with LVESV decreasing to 58.2 mL and LVEDV to 110 mL, together with reductions in left ventricular dimensions. PASP decreased to 32 mmHg, and NT-proBNP levels declined to 7261.7 pg/mL. Blood pressure improved, serum potassium remained stable at 4.95 mmol/L, and no severe hyperkalemia, intradialytic hypotension, post-dialysis hypotension, or heart failure-related hospitalization occurred; **Conclusions:** In this case series, SV was associated with improved cardiac function, reverse remodeling, pulmonary pressure reduction, and an acceptable safety profile in patients with HFrEF undergoing MHD. These findings suggest a potential therapeutic role for SV in selected dialysis patients and support further investigation.

## 1. Introduction and Clinical Significance

End-stage kidney disease (ESKD) affects a large number of people in Europe and worldwide. In 2023 in Europe, approximately 78.000 patients required kidney replacement therapy, of whom 83% started hemodialysis [1].

Patients undergoing maintenance hemodialysis (MHD) present a 20-fold higher risk of cardiovascular disease (CVD) compared to the general population [2].

Nearly one-third of the ESKD patients undergoing MHD suffer from heart failure (HF) [3].

CVD and HF are highly prevalent within the dialysis population. Both incidence and prevalence increase with age and in the presence of risk factors such as hypertension, diabetes mellitus, and dyslipidemia. In addition, the uremic milieu contributes to an accelerated atherosclerosis process through mechanisms including chronic inflammation, endothelial dysfunction, and oxidative stress [3,4].

Over the past two decades, the use of angiotensin-converting enzyme inhibitors (ACE inhibitors) and angiotensin receptor blockers (ARBs) in dialysis patients has been associated with improved cardiovascular outcomes. Observational studies have suggested that long-term therapy with ACE inhibitors or ARBs may reduce all-cause mortality and cardiovascular events, particularly HF, in both MHD and peritoneal dialysis populations [5,6,7].

Despite advances in medical therapy and growing evidence from clinical trials and observational studies in MHD patients, HF continues to be associated with substantial morbidity and mortality, increased hospitalization risk, and heightened susceptibility to treatment-related complications [4].

Sacubitril/valsartan (SV), an angiotensin receptor–neprilysin inhibitor, was approved as a treatment for HF for the first time in 2015 [8] and is currently considered a cornerstone therapy in contemporary HF management guidelines [9].

SV exerts its effects through a dual mechanism. Sacubitril inhibits neprilysin, which is responsible for the degradation of several vasoactive peptides, including atrial natriuretic peptide (ANP), B-type natriuretic peptide (BNP), C-type natriuretic peptide (CNP), adrenomedullin, endothelin, bradykinin, and substance P, as well as angiotensin I and II. Neprilysin has a higher affinity for ANP than BNP; consequently, ANP levels increase rapidly after sacubitril administration, while BNP may initially rise and subsequently decrease as left ventricular wall stress is reduced. Elevated ANP levels promote cyclic guanosine monophosphate (cGMP) production in vascular smooth muscle cells, resulting in vasodilation, natriuresis, and inhibition of fibrosis [10].

Valsartan, an angiotensin II receptor blocker (ARB), selectively antagonizes the angiotensin II type 1 (AT1) receptor, thereby inhibiting the renin–angiotensin–aldosterone system (RAAS). This leads to reduced vasoconstriction, decreased aldosterone secretion, attenuation of sympathetic nervous system activation, and inhibition of sodium and water retention.

The combined action of sacubitril and valsartan results in enhanced vasodilation, suppression of maladaptive neurohormonal activation, and, over time, reduction in cardiac fibrosis, hypertrophy, and adverse ventricular remodeling [11,12].

SV has been shown to reduce the risk of cardiovascular death and HF hospitalization and to improve symptoms in patients with chronic HF with reduced ejection fraction (HFrEF) when compared with ACEi or ARBs [13]. This combination therapy acts synergistically by promoting natriuresis and diuresis, inducing vasodilation, and preventing maladaptive cardiac remodeling [14].

However, the efficacy and safety of SV in patients undergoing MHD remain unclear, since this population was excluded from the PARADIGM-HF trial [11] due to safety concerns. Consequently, evidence supporting the use of SV in dialysis patients is still limited, despite the markedly increased cardiovascular risk characterizing this population.

We therefore report a case series describing the clinical and hemodynamic effects of SV affected by HFrEF undergoing MHD.

## 2. Case Presentation

We present data from four hemodialysis patients with HFrEF who were treated with SV. Before starting SV, all patients were treated with ACEIs or ARBs.

A low dose of SV was introduced at the moment on HF diagnosis, stopping ACEi or ARB treatment. Daily dose-up titration was made based on tolerability, intended as blood pressure trend and serum potassium level.

We compared the data before the start of SV treatment and 3, 6, and 12 months later, including post-dialysis blood pressure; post-dialysis transthoracic echocardiography, when body weight reached dry weight (DW) to assess cardiac parameters, such as the ejection fraction (EF), left arial dimension (LAD), left ventricular end-systolic volume (LVESV), left ventricular end-diastolic volume (LVEDV), left ventricular internal diameter in diastole (LVIDD), left ventricular internal diameter in systole (LVIDS), interventricular septum diameter (IVSd) and pulmonary artery systolic pressure (PASP).

Furthermore, we evaluate N-terminal pro-brain natriuretic peptide (NT-pro BNP), hemoglobin, potassium, and parathormone trends. All laboratory data were collected pre-dialysis, on the first hemodialysis session of the week (summary patient data are shown on Table 1).

DW was evaluated by performing chest X-ray after dialysis on the last hemodialysis session of the week; clinical findings were as follows: no edema and/or dyspnea, proper blood pressure. All patients received counseling regarding sodium and fluid intake as part of standard hemodialysis management.

During the follow-up, we did not change any of their medications related to HF except for SV; we did not change the doses or modality of hemodialytic treatment. Blood pressure target range was supposed to be lower than 135 mmHg (systolic blood pressure) post-dialysis. Antihypertensive medicines were revisited, when necessary, to prevent hypotension. During the treatment, we monitored adverse reactions such as hyperkaliemia, defined as pre-dialysis potassium serum levels exceeding 5.5 mmol/L, and hypotension, established as systolic blood pressure < 110 mmHg before and during dialysis.

Given the small sample size and the exploratory nature of this case series, the analysis was primarily descriptive. Continuous variables are reported as median, and temporal changes are presented descriptively. Percentage changes from baseline were calculated to illustrate the magnitude of change during follow-up.

All the patients were informed of the off-label use of SV treatment, and they were closely monitored from both a cardiological and nephrological perspective.

### 2.1. Baseline Characteristics

The demographic, clinical, laboratory, and echocardiographic characteristics of the study population are summarized in Table 1.

All patients were male, with a mean age of 71.7 years. The mean dialysis vintage was 32.3 months. All patients were receiving maintenance hemodialysis three times weekly, with each session lasting four hours.

The mean systolic blood pressure (SBP) and diastolic blood pressure (DBP) at baseline were 147.5 mmHg and 72.5 mmHg, respectively.

All patients had a history of long-standing hypertension and coronary artery disease, while two patients had long-standing type 2 diabetes mellitus. The underlying causes of ESKD were diabetic nephropathy in two patients and hypertensive/renovascular nephropathy in the remaining two. Despite differences in age and kidney disease etiology, all patients presented with advanced HF characterized by severely reduced left ventricular systolic function and marked left ventricular remodeling at baseline. The severity of cardiac dysfunction likely reflected the cumulative impact of multiple cardiovascular risk factors, including long-standing hypertension, coronary artery disease, long-standing diabetes mellitus when present, and the cardiovascular burden associated with ESKD requiring MHD.

Baseline echocardiographic evaluation showed a mean left ventricular ejection fraction (LVEF) of 35.5%, left ventricular end-systolic volume (LVESV) of 118.5 mL, left ventricular end-diastolic volume (LVEDV) of 185.7 mL, left ventricular internal diameter in diastole (LVIDD) of 61.5 mm, left ventricular internal diameter in systole (LVIDS) of 48.2 mm, interventricular septal thickness in diastole (IVSd) of 14.2 mm, left atrial diameter (LAD) of 49.2 mm, and pulmonary artery systolic pressure (PASP) of 52.7 mmHg.

All patients presented baseline NT-proBNP levels >35,000 pg/mL, corresponding to the upper quantification limit of the assay used at our institution.

Baseline laboratory parameters included serum potassium of 4.85 mmol/L, hemoglobin of 11.12 g/dL, and parathyroid hormone (PTH) levels of 270.25 pg/mL.

At baseline, all patients were classified as New York Heart Association (NYHA) functional class, two patients presented as NYHA class III and the other two as class IV.

### 2.2. Follow-Up Results

During the 12-month follow-up period, progressive improvements were observed in blood pressure control, cardiac function, and echocardiographic parameters, with evidence of reverse cardiac remodeling, including increased LVEF and reductions in ventricular volumes, LAD, and PASP, as shown in Table 2.

#### 2.2.1. Blood Pressure

**Figure 1:**
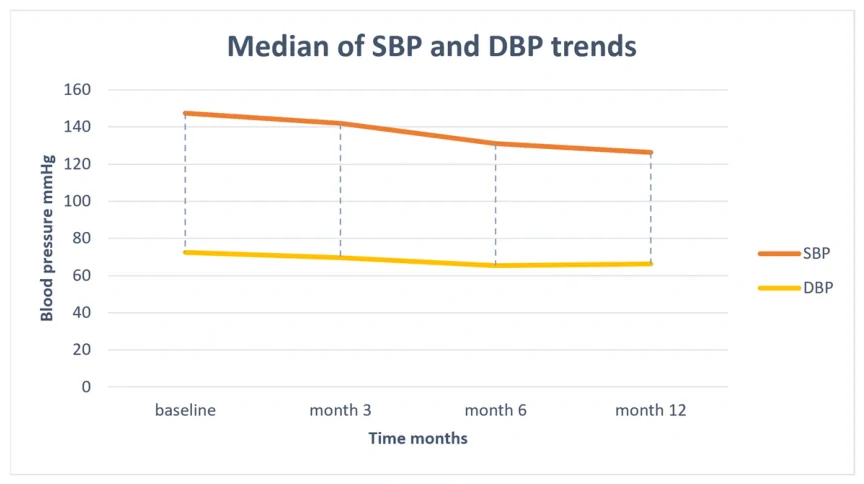
Median of systolic and diastolic blood pressure trends.

Systolic and diastolic blood pressure showed a progressive and sustained reduction over time. Mean SBP decreased from 147.5 mmHg at baseline to 142 mmHg at 3 months, 131 mmHg at 6 months, and 126.2 mmHg at 12 months. A similar trend was observed for DBP, which decreased from 72.5 mmHg at baseline to 69.5 mmHg, 65.2 mmHg, and 66.2 mmHg at 3, 6, and 12 months, respectively (Figure 1).

#### 2.2.2. Left Ventricular Systolic Function and Volumes

**Figure 2:**
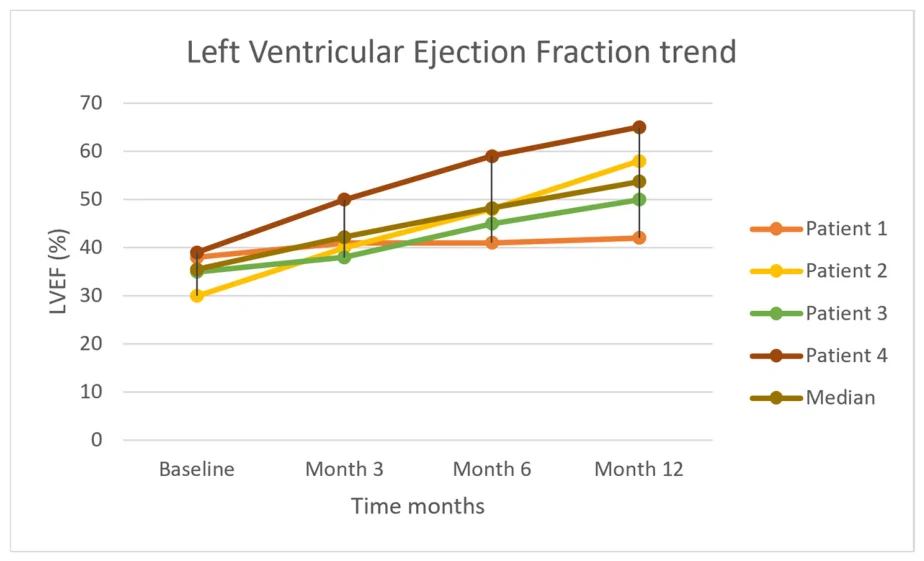
Left ventricular ejection fraction trend reported for single patients and median.

Left ventricular systolic function progressively improved over time. LVEF increased from 35.5% at baseline to 42.2% at 3 months, 48.2% at 6 months, and 53.7% at 12 months, as shown in Figure 2.

**Figure 3:**
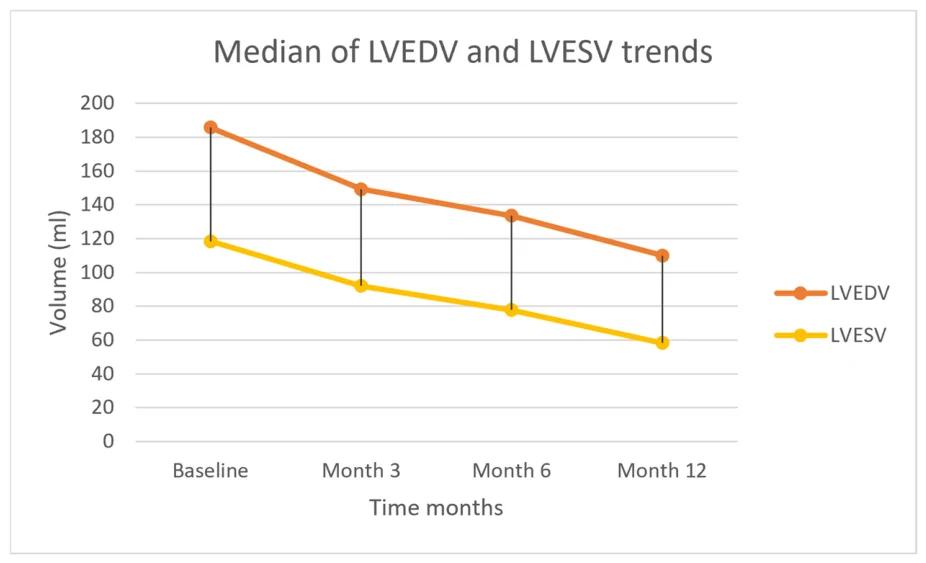
Median of left ventricular end-diastolic volume, LVEDV; left ventricular end-systolic volume, LVESV.

This improvement was accompanied by a progressive reduction in left ventricular volumes. LVEDV decreased from 185.7 mL at baseline to 149.2 mL, 133.5 mL, and 110 mL at 3, 6, and 12 months, respectively. Similarly, LVESV decreased from 118.5 mL to 92 mL, 77.7 mL, and 58.2 mL over the same time points. Figure 3 shows LVEDV and LVESV trends.

#### 2.2.3. Cardiac Structural Parameters

Progressive reverse remodeling was observed in cardiac dimensions. LVIDD decreased from 61.5 mm at baseline to 56.7 mm, 55.5 mm, and 49.5 mm at 3, 6, and 12 months, respectively. LVIDS showed a reduction from 48.2 mm to 47 mm, 39.7 mm, and 31.5 mm over follow-up.

LAD decreased progressively from 49.2 mm at baseline to 40.5 mm, 39.7 mm, and 36 mm at 3, 6, and 12 months, respectively. Interventricular septal thickness also showed a mild reduction from 14.2 mm to 13 mm, 12.7 mm, and 12.5 mm.

**Figure 4:**
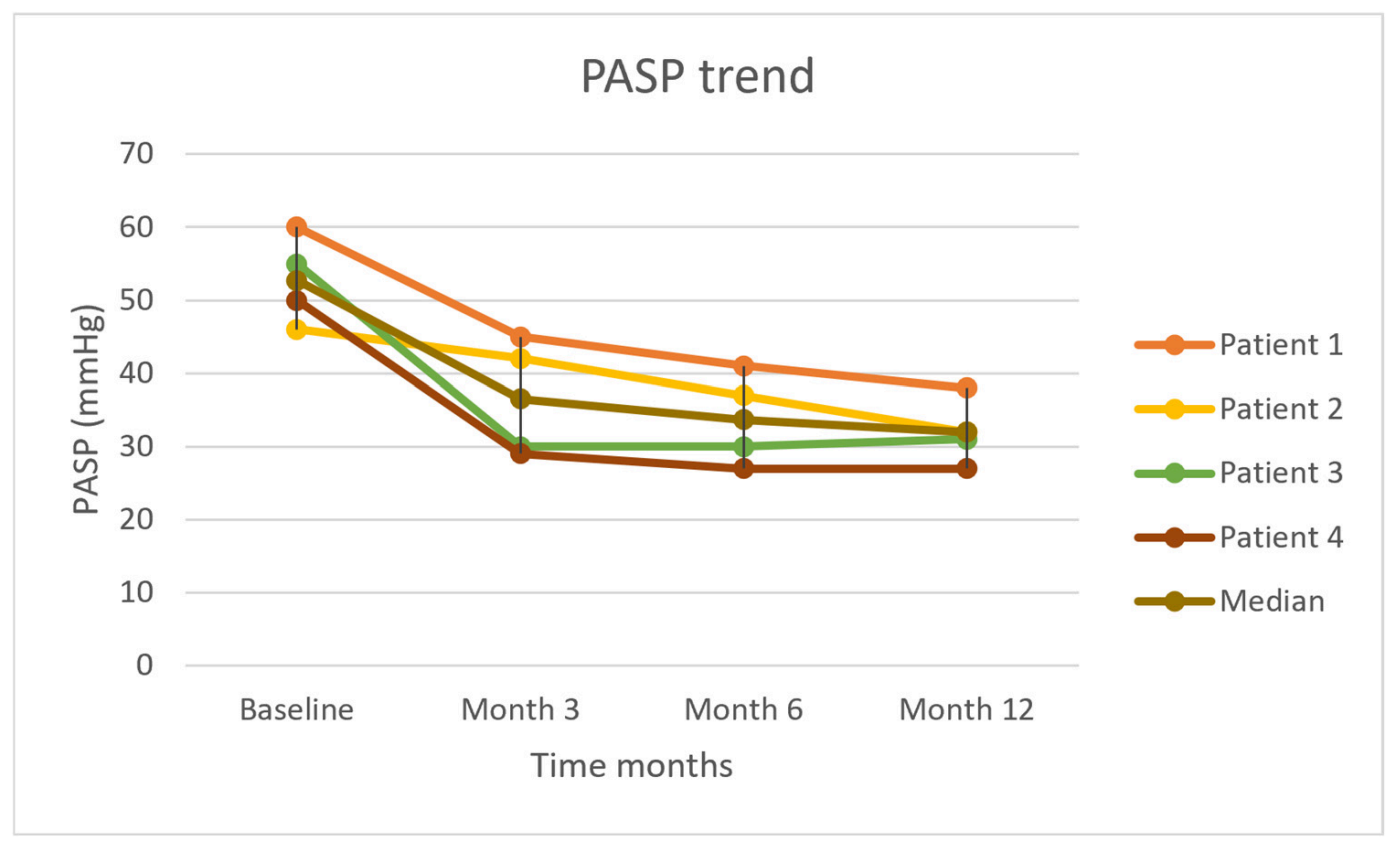
Pulmonary artery systolic pressure, PASP trends, reported for single patients and median.

PASP decreased from 52.7 mmHg at baseline to 36.5 mmHg, 33.7 mmHg, and 32.0 mmHg at 3, 6, and 12 months, respectively, as shown in Figure 4.

#### 2.2.4. NT-proBNP

**Figure 5:**
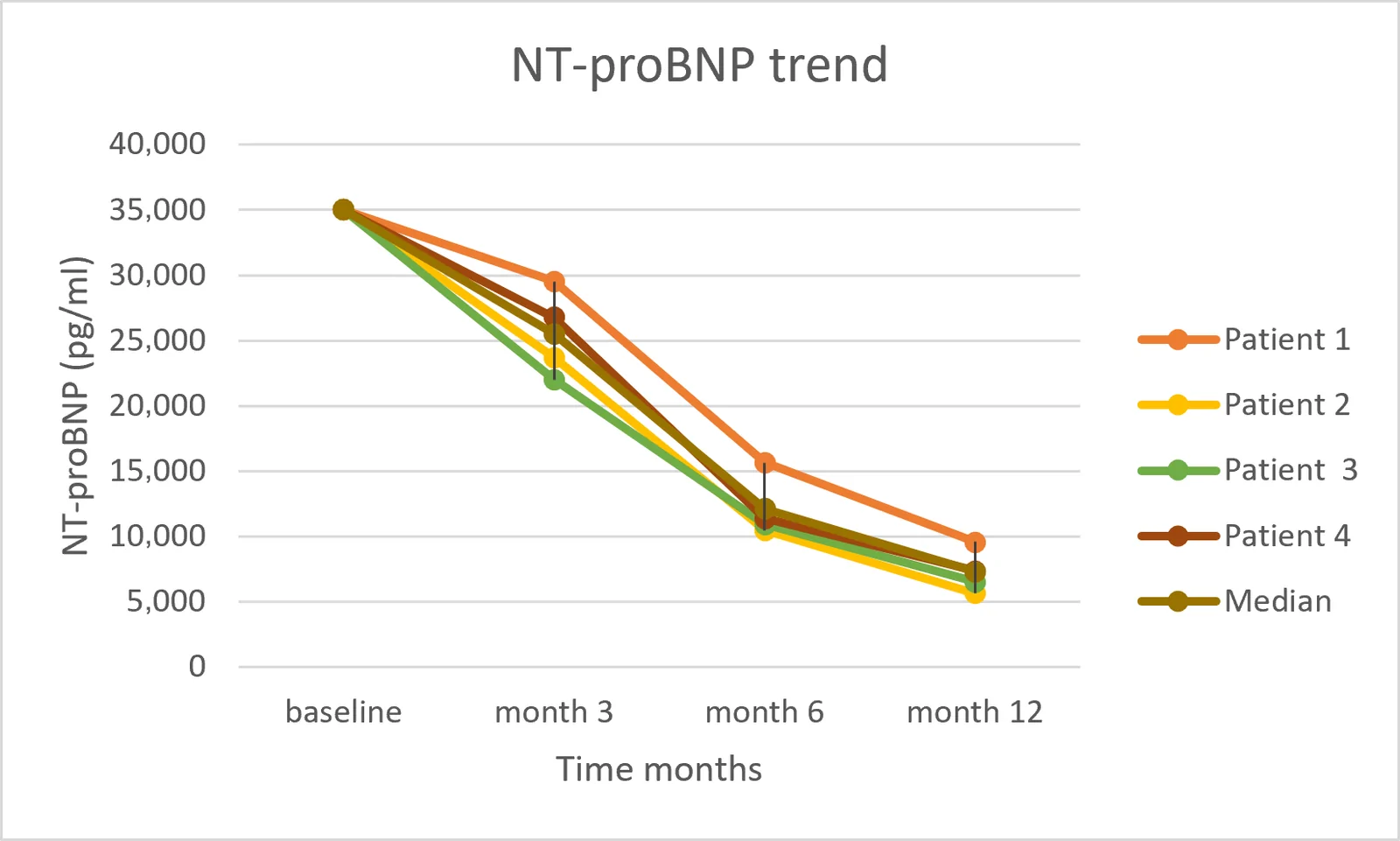
N-terminal pro-brain natriuretic peptide, NT-proBNP trends, reported for single patients and median.

NT-proBNP levels showed a marked progressive reduction throughout follow-up. Values decreased from >35,000 pg/mL at baseline (upper assay limit) to 25,492.7 pg/mL at 3 months, 12,095.5 pg/mL at 6 months, and 7261.7 pg/mL at 12 months, indicating a sustained reduction in cardiac wall stress. The NT-proBNP trend is shown in Figure 5.

#### 2.2.5. Laboratory Parameters

Serum potassium, hemoglobin, and parathyroid hormone levels remained overall stable throughout follow-up, with only minor fluctuations within normal variability ranges.

#### 2.2.6. Functional Status

At baseline, two patients were classified as NYHA class III and two as NYHA class IV, indicating advanced functional impairment.

At one year of timeframe observation, an improvement in NYHA class was registered; one patient presented as NYHA Class I and the others as NYHA Class II.

During the follow-up period, we did not observe variation in 24 h residual urine output in all of the patients.

All detailed data regarding all single patients are summarized in Table 3.

## 3. Discussion

In this case series, we describe our single-center experience with SV in four patients undergoing maintenance hemodialysis with concomitant HFrEF. Treatment was associated with favorable changes in cardiac structure and function, a marked reduction in NT-proBNP levels, improved blood pressure control, and a reassuring safety profile, with no episodes of hyperkalemia or symptomatic hypotension observed during follow-up.

SV is currently considered one of the four cornerstone therapies for HFrEF, alongside β-blockers, mineralocorticoid receptor antagonists, and sodium–glucose cotransporter-2 inhibitors [15]. Despite its established efficacy in the general HFrEF population, patients with advanced chronic kidney disease CKD and ESKD requiring dialysis were largely excluded from the pivotal clinical trials, resulting in limited evidence regarding its efficacy and safety in this high-risk population [16].

Given the high prevalence of HF and its associated adverse outcomes [4,17] in patients with ESKD undergoing hemodialysis, optimal management remains a major clinical challenge. Emerging evidence regarding the use of SV in this population is encouraging, particularly among patients with HFrEF [18].

In our cohort, treatment with SV was associated with progressive reverse cardiac remodeling, characterized by improvements in left ventricular systolic function and reductions in ventricular volumes over the 12-month follow-up period. These findings are consistent with a growing body of evidence supporting the beneficial effects of SV on cardiac structure and function in patients undergoing maintenance hemodialysis. Niu et al. reported a marked improvement in LVEF from 31.3% to 45.1% following SV therapy in 26 hemodialysis patients with HFrEF [19]. Similar results were observed by Wen et al. in a larger cohort of 54 patients [20], while Wang et al. demonstrated significant improvements in LVEF from 35.1% to 49.8% after 12 months of treatment in an observational study involving 110 MHD patients [21]. More recently, a meta-analysis by Nguyen et al., including 22 studies, confirmed a significant improvement in LVEF, particularly among patients with HFrEF receiving maintenance hemodialysis [22]. Collectively, these findings support the hypothesis that the cardioprotective effects of SV extend beyond the non-dialysis population and may contribute to meaningful reverse remodeling in patients with ESKD.

In addition to improvements in left ventricular systolic function and ventricular volumes, we observed a marked reduction in pulmonary artery systolic pressure (−39.4%) and left atrial diameter (−26.8%). Left atrial enlargement is a recognized marker of chronically elevated left ventricular filling pressures and adverse cardiovascular outcomes. Therefore, the observed reduction in LAD may reflect improved diastolic filling pressures and reduced chronic volume overload. Together with the decrease in PASP, these findings further support the occurrence of reverse cardiac remodeling and improved hemodynamic status during SV therapy [23,24,25].

A marked reduction in NT-proBNP levels was also observed throughout follow-up. Although interpretation of natriuretic peptides in dialysis patients remains challenging because of reduced renal clearance, serial changes continue to provide valuable information regarding myocardial wall stress and cardiovascular risk. Our findings are consistent with those reported by Mapelli et al., Ding et al., and Wang et al., all of whom demonstrated significant reductions in NT-proBNP levels following SV initiation in hemodialysis patients [21,26,27]. Furthermore, Cong et al. reported a greater reduction in NT-proBNP levels with SV compared with ARB therapy alone, supporting the additional benefits associated with neprilysin inhibition [25]. Taken together, these observations suggest that SV may contribute to improved neurohormonal and hemodynamic status in this patient population.

SV therapy was associated with improved blood pressure control in our patients. Median SBP decreased from 147.5 mmHg to 126.25 mmHg, and DBP from 72.5 mmHg to 66.2 mmHg. The British HARP-III trial demonstrated that SV reduced systolic and diastolic blood pressure by 5.4 mmHg and 2.1 mmHg, respectively, in patients with CKD. Similarly, studies by Ding et al. and Wen et al. reported improved blood pressure control following SV initiation in hemodialysis patients [27]. These effects likely reflect the dual antihypertensive action of SV, combining RAAS inhibition with enhanced natriuretic peptide activity. Consequently, SV may allow for a reduction in the number of antihypertensive agents required.

Regarding safety, SV was well tolerated throughout the follow-up period. No episodes of intradialytic or post-dialysis hypotension were observed, and serum potassium levels remained stable, with no patient developing severe hyperkalemia. Notably, two patients were already receiving potassium binders because of previous hyperkalemia during treatment with ACE inhibitors, ARBs, and/or mineralocorticoid receptor antagonists. Switching to SV did not require any adjustment in sodium zirconium cyclosilicate dosage, further supporting the favorable safety profile of this therapy in our cohort.

These findings are consistent with the available literature. A recent meta-analysis including 26 studies and 2494 dialysis patients with heart failure reported that SV was generally well tolerated and was not associated with an increased risk of severe hyperkalemia or symptomatic hypotension [22]. While the efficacy and safety of SV are well established in the general HFrEF population, evidence in patients with ESKD remains limited [13]. Nevertheless, observational studies have reported favorable effects on cardiovascular outcomes, including reductions in HF rehospitalizations and improvements in cardiac function, without significant adverse effects on serum potassium levels or residual renal function [28].

In contrast, Nguyen et al. reported only a marginal benefit in all-cause mortality and no significant reduction in HF hospitalizations [22]. The attenuated natriuretic and diuretic response observed in dialysis patients may explain the smaller clinical benefits reported compared with non-dialysis HFrEF populations, particularly regarding heart failure-related rehospitalizations [29].

In our series, no patient required rehospitalization for heart failure during follow-up. Moreover, one patient experienced sufficient improvement in cardiac function and hemodynamic status to allow removal of the implantable cardioverter–defibrillator and subsequent kidney transplantation. Two additional patients remained free from hospitalization throughout the observation period, whereas one patient died from severe sepsis, an event considered unrelated to SV therapy.

Taken together, our findings suggest that SV may represent a safe and potentially effective therapeutic option for selected hemodialysis patients with HFrEF, with favorable effects on cardiac remodeling, biomarker profiles, and blood pressure control. However, given the small sample size and observational nature of the available evidence, the impact of SV on HF hospitalizations, cardiovascular mortality, and overall survival remains uncertain. Larger prospective studies and randomized controlled trials are needed to better define the role of SV in this high-risk population.

## 4. Conclusions

In this case series of patients undergoing maintenance hemodialysis with concomitant HFrEF, treatment with SV was associated with favorable changes in cardiac structure and function, including evidence of reverse cardiac remodeling, improved blood pressure control, and a marked reduction in NT-proBNP levels. Moreover, SV was well tolerated, with no episodes of symptomatic hypotension or severe hyperkalemia observed during follow-up.

Although limited by the small sample size and observational design, our findings add to the growing body of evidence supporting the potential role of SV in the management of HFrEF among patients receiving maintenance hemodialysis. Larger prospective studies and randomized controlled trials are needed to confirm these observations and to clarify the impact of SV on HF hospitalizations, cardiovascular outcomes, and survival in this high-risk population.

## Figures and Tables

**Table 1:** Baseline characteristics: CKD (chronic kidney disease), AVF (arteriovenous fistula), CVC (central venous catheter), AFB-K (acetate-free biofiltration), OL-HDF POST (online hemodiafiltration post-dilution), HFHD (high-flux hemodialysis), ICD (implantable cardioverter–defibrillator), LVEF (left ventricular ejection fraction), LVEDV (left ventricular end-diastolic volume), LVESV (left ventricular end-systolic volume); LVIDD (left ventricular internal diameter in diastole), LVIDS (left ventricular internal diameter in systole), IVSd (interventricular septum), LAD (left atrial diameter), PASP (pulmonary artery systolic pressure), ACE-I (angiotensin-converting enzyme inhibitor), ARB (angiotensin receptor blocker), MRA (mineralocorticoid receptor antagonist), SZC (sodium zirconium cyclosilicate).

	Patient 1	Patient 2	Patient 3	Patient 4
**Age (y)**	78	46	73	90
**Gender**	M	M	M	M
**Cause of CKD**	Diabetes	Diabetes	Hypertension/ Renovascular	Hypertension/ Renovascular
**Dialysis duration (m)**	45	19	40	25
**Vascular access**	AVF	AVF	AVF	CVC
**Dialysis**	AFB-K	OL-HDF post	OL-HDF post	HFHD
**Number of dialysis/week (** ***n*** **)**	3	3	3	3
**Session duration (h)**	4	4	4	4
**Kt/V**	1.7	1.3	1.6	1.5
**Blood pressure pre** **-** **dialysis (mmHg)**	120/50	155/75	155/80	160/85
**Heart rate (b/min)**	87	68	75	54
**NYHA Class**	4	3	4	4
**Medical history**				
**Hypertension**	YES	YES	YES	YES
**Coronary artery disease**	YES	YES	YES	YES
**Atrial fibrillation**	YES	NO	NO	NO
**ICD**	NO	YES	NO	NO
**Diabetes**	YES	YES	NO	NO
**Peripheral vasculopathy**	YES	NO	NO	NO
**Residual diuresis (ml/24 h)**	<300	500	600	800
**Medications**				
**ACE-I or ARB therapy**	YES	YES	YES	YES
**MRA**	YES	YES	NO	NO
**Beta-blocker**	YES	YES	YES	YES
**Diuretic**	NO	YES	YES	YES
**Calcium blocker**	YES	YES	YES	YES
**Alpha-lytic**	YES	YES	YES	YES
**Aspirin/clopidogrel**	YES/NO	NO/YES	YES/YES	YES/NO
**SZC therapy**	NO	YES	NO	YES
**Mean laboratory data**				
**Potassium (mmol/L)**	4.5	4.8	4.7	5.4
**Sodium (mmol/L)**	137	141	139	137
**Calcium (mg/dL)**	8.8	8.7	9	9.2
**Phosphate (mg/dL)**	3.9	5.7	5	3.5
**Parathormone (pg/mL)**	240	328	163	350
**Hemoglobin (g/dL)**	11.3	11.2	10.9	11.1
**Creatinine (mg/dL)**	8.32	9.88	9.24	11.7
**Urea (mg/dL)**	108	153	139	174
**Cardiological parameters**				
**NT-proBNP (pg/mL)**	>35,000	>35,000	>35,000	>35,000
**LVEF (%)**	38	30	35	39
**LVEDV (mL)**	168	175	189	211
**LVESV (mL)**	96	120	124	134
**LVIDD (mm)**	54	70	62	60
**LVIDS (mm)**	43	50	46	54
**IVSd (mm)**	12	15	14	16
**LAD (mm)**	45	50	49	53
**PASP (mmHg)**	60	46	55	50

The mean follow-up duration was 12 months.

**Table 2:** Results expressed as median and percentage changes from baseline. Systolic blood pressure, SBP; diastolic blood pressure, DBP; left ventricular ejection fraction, LVEF; left ventricular end-diastolic volume, LVEDV; left ventricular end-systolic volume, LVESV; left ventricular end-diastolic diameter, LVIDD; left ventricular internal diameter in diastole, LVIDS; left ventricular internal diameter in systole, LVIDD; left ventricular internal diameter in systole, LVIDS; interventricular septum, IVSd; left atrial diameter, LAD; pulmonary artery systolic pressure, PASP; N-terminal pro-brain natriuretic peptide, NT-proBNP.

	Baseline	3 Months	6 Months	12 Months	% Variation
**SBP**	147.5	142	131	126.2	−14.4%
**DBP**	72.5	69.5	65.2	66.2	−8.7%
**LVEF**	35.5	42.2	48.2	53.7	+51.4%
**LVEDV**	185.7	149.2	133.5	110	−40.8%
**LVESV**	118.5	92	77.7	58.2	−50.8%
**LVIDD**	61.5	56.7	55.5	49.5	−19.5%
**LVIDS**	48.2	47	39.7	31.5	−34.6%
**IVSd**	14.2	13	12.7	12.5	−12%
**LAD**	49.2	40.5	39.7	36	−26.8%
**PASP**	52.7	36.5	36.7	32	−39.4%
**NT-proBNP**	>35,000	25,492.7	12,095.5	7261.7	−79.3%

**Table 3:** N-terminal pro-brain natriuretic peptide, NT-proBNP; left ventricular ejection fraction, LVEF; left ventricular end-diastolic volume, LVEDV; left ventricular end-systolic volume, LVESD; left ventricular end-systolic diameter LVIDD; left ventricular internal diameter in systole, LVIDS; interventricular septum, IVSd; left atrial diameter, LAD; pulmonary artery systolic pressure, PASP; systolic blood pressure, SBP; diastolic blood pressure, DBP.

Variable	Time	Patient 1	Patient 2	Patient 3	Patient 4
**Starting dose**	Baseline	24/26 ½ tablet BID	24/26 mg BID	24/26 mg BID	24/26 ½ tablet BID
**Maximum tolerated dose**	During follow-up	24/26 mg BID	49/51 mg BID	24/26 mg BID	24/26 mg BID
**Follow-up (months)**		12	12	12	12
**HF hospitalization (n)**		0	0	0	0
**NT-proBNP (pg/mL)**	Month 3	29,521	23,678	21,987	26,785
	Month 6	15,657	10,450	10,923	11,352
	Month 12	9563	5627	6512	7345
**LVEF (%)**	Month 3	41	40	38	50
	Month 6	41	48	45	59
	Month 12	42	58	50	65
**LVEDV/LVESD (mL)**	Month 3	128/83	163/103	179/114	127/68
	Month 6	117/76	145/86	159/96	113/53
	Month 12	108/71	134/69	97/46	101/47
**LVIDD/LVIDS (mm)**	Month 3	53/41	65/51	57/50	52/46
	Month 6	51/41	61/50	58/39	52/29
	Month 12	51/39	49/28	46/33	52/26
**IVSd (mm)**	Month 3	12	11	13	16
	Month 6	13	11	13	14
	Month 12	13	11	13	13
**LAD (mm)**	Month 3	34	35	47	46
	Month 6	34	35	46	44
	Month 12	32	37	34	41
**PASP (mmHg)**	Month 3	45	42	30	29
	Month 6	41	37	30	27
	Month 12	38	32	31	27
**SBP (mmHg)**	Month 3	123	145	153	147
	Month 6	118	136	135	135
	Month 12	120	130	125	130
**DBP (mmHg)**	Month 3	55	80	76	67
	Month 6	50	75	71	65
	Month 12	52	80	70	63
**Potassium (mmol/L)**	Month 3	5.0	4.2	5.0	3.7
	Month 6	4.9	5.1	5.0	4.7
	Month 12	5.0	5.1	5.1	4.6
**Hemoglobin (g/dL)**	Month 3	13.0	11.5	13.0	11.2
	Month 6	11.0	12.5	10.7	11.6
	Month 12	11.3	11.0	11.1	11.4
**Parathyroid hormone (pg/mL)**	Month 3	232	380	183	270
	Month 6	186	230	281	340
	Month 12	164	220	238	287

## Data Availability

The original contributions presented in this study are included in the article. Further inquiries can be directed to the corresponding author.
